# Cytotoxic Potential of the Novel Horseshoe Crab Peptide Polyphemusin III

**DOI:** 10.3390/md16120466

**Published:** 2018-11-26

**Authors:** Mariana B. Marggraf, Pavel V. Panteleev, Anna A. Emelianova, Maxim I. Sorokin, Ilia A. Bolosov, Anton A. Buzdin, Denis V. Kuzmin, Tatiana V. Ovchinnikova

**Affiliations:** 1 M.M. Shemyakin & Yu.A. Ovchinnikov Institute of Bioorganic Chemistry, the Russian Academy of Sciences, Mikhluho-Maklaya str. 16/10, Moscow 117997, Russia; thpcb92@mail.ru (M.B.M.); alarm14@gmail.com (P.V.P.); annaemelyan@gmail.com (A.A.E.); b_off2@mail.ru (I.A.B.); buzdin@oncobox.com (A.A.B.); denisk@list.ru (D.V.K.); 2 Department of Bioinformatics and Molecular Networks, Omicsway Corp., Walnut, CA 91789, USA; sorokin@oncobox.com; 3 Department of Biotechnology, I.M. Sechenov First Moscow State Medical University (Sechenov University), Moscow 119991, Russia

**Keywords:** antimicrobial peptide, cytotoxicity, β-hairpin, polyphemusins, tachyplesins, cell death, signaling pathways

## Abstract

Biological activity of the new antimicrobial peptide polyphemusin III from the horseshoe crab *Limulus polyphemus* was examined against bacterial strains and human cancer, transformed, and normal cell cultures. Polyphemusin III has the amino acid sequence RRGCFRVCYRGFCFQRCR and is homologous to other β-hairpin peptides from the horseshoe crab. Antimicrobial activity of the peptide was evaluated and MIC (minimal inhibitory concentration) values were determined. IC_50_ (half-maximal inhibitory concentration) values measured toward human cells revealed that polyphemusin III showed a potent cytotoxic activity at concentrations of <10 μM. Polyphemusin III caused fast permeabilization of the cytoplasmic membrane of human leukemia cells HL-60, which was measured with trypan blue exclusion assay and lactate dehydrogenase-release assay. Flow cytometry experiments for annexin V-FITC/ propidium iodide double staining revealed that the caspase inhibitor, Z-VAD-FMK, did not abrogate disruption of the plasma membrane by polyphemusin III. Our data suggest that polyphemusin III disrupts the plasma membrane integrity and induces cell death that is apparently not related to apoptosis. In comparison to known polyphemusins and tachyplesins, polyphemusin III demonstrates a similar or lower antimicrobial effect, but significantly higher cytotoxicity against human cancer and transformed cells in vitro.

## 1. Introduction

Antimicrobial peptides (AMPs) are important components of the innate host defense in many organisms. AMPs exhibit activity against pathogenic bacteria, fungi, viruses, and protozoans [1,2,3]. Some antimicrobial peptides are also considered as putative anticancer agents [4,5,6]. Previous studies demonstrated that β-hairpin cationic antimicrobial peptides, for example, tachyplesin I from horseshoe crab hemocytes, gomesin from spider hemocytes, and protegrin-1 from porcine leukocytes displayed both antibacterial and antitumor activities [7,8,9,10].

Polyphemusins are family of β-hairpin cationic antimicrobial peptides that play a role in the innate immunity of horseshoe crabs. They were isolated from hemocytes of the horseshoe crab *Limulus polyphemus* [11]. These peptides are structurally close to another family of horseshoe crab antimicrobial peptides, tachyplesins, isolated from the species *Tachypleus trindentatus, Tachypleus gigas*, and *Carcinoscorpius rotundicauda.* Polyphemusins and tachyplesins polypeptide chains consist of 18 and 17 amino acid residues, respectively, and contain two disulfide bonds. The peptides from both groups have a high net positive charge due to several arginine and lysine residues in their amino acid sequences [11,12,13]. Polyphemusins and tachyplesins can disrupt both outer and inner membranes of Gram-negative bacteria [14,15,16]. Cationic and amphipathic properties of polyphemusins and tachyplesins have been implicated as the most essential features for the mode of their action towards microorganisms [14,16,17]. It has been shown that these peptides selectively interact with negatively charged phospholipids of bacterial membranes [14,18].

Similarly to tachyplesins, polyphemusins also exhibit a broad spectrum of biological activities. Naturally occurring and synthetic polyphemusin I, polyphemusin II, and their analogs inhibit growth of both Gram-positive and Gram-negative bacteria, as well as some fungi at submicromolar and micromolar concentrations [11,14,16,19], mammalian tumor cells at micromolar concentrations [8,9], have a high affinity for lipopolysaccharides [11,14], and may cause degradation of *Staphylococcus aureus* biofilms [20].

So far, five β-hairpin peptides (polyphemusin I, polyphemusin II, tachyplesin I, tachyplesin II, tachyplesin III) have been isolated from the four above-mentioned species of horseshoe crabs and only for two of them, tachyplesin I and tachyplesin II, have the precursor nucleotide and amino acid sequences been reported [21]. The complete coding sequences of prepropolyphemusins were obtained by using the preprotachyplesin I sequential blasting in the *Limulus polyphemus* genome database. Interestingly, the gene encoding polyphemusin II was not identified in this database. Instead, we identified the novel isoform named polyphemusin III (PM III). PM III has a molecular mass of 2309.09 Da and the amino acid sequence RRGCFRVCYRGFCFQRCR including six basic arginine residues, providing a net positive charge of +6.

We expressed the recombinant PM III in *Escherichia coli* and investigated cytotoxic properties of polyphemusins against seven bacterial strains, both Gram-positive and Gram-negative, as well as towards four human cancer cell lines and one transformed human cell line. In addition, two types of normal human primary cell cultures were used to determine the peptides’ cytotoxicity. We also compared the biological properties of PM III with those of the other two isoforms—polyphemusin I (PM I), polyphemusin II (PM II), and with tachyplesins—tachyplesin I (TP I), tachyplesin II (TP II), and tachyplesin III (TP III).

PM III demonstrated a high cytotoxicity at concentrations of <10 µM. Compared to tachyplesins and other polyphemusins, PM III had higher cytotoxic activities for human cells. In contrast, PM III showed lower antibacterial activity compared to tachyplesins, PM I, and PM II. A cytotoxic effect of PM III was observed after 15 min of incubation without further increase over time. The cell death promoting mechanism presumably was not associated with the caspase-dependent apoptosis, as the disruption of plasma membrane integrity was not abrogated by the caspase inhibitor, Z-VAD-FMK.

## 2. Results

### 2.1. Identificantion of Antimicrobial Peptide

**Figure 1:**
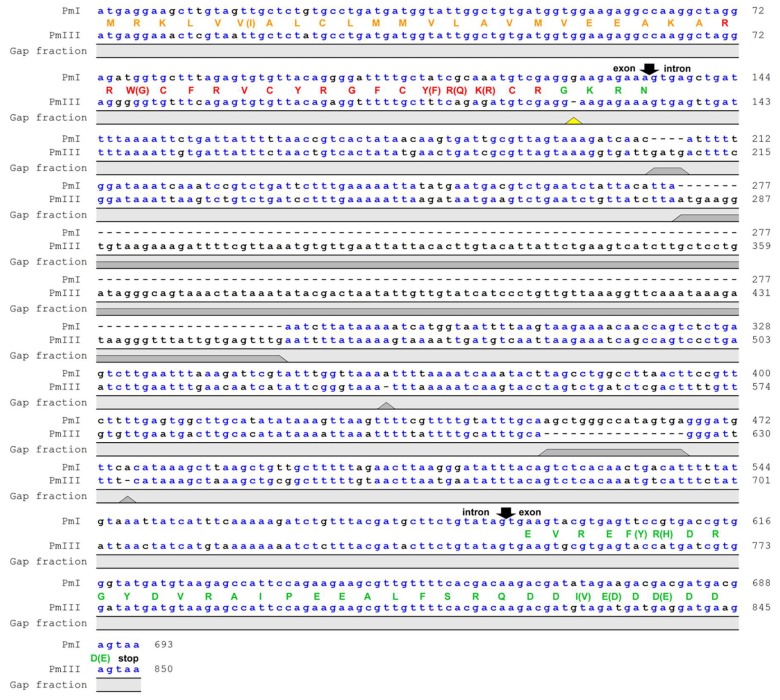
Nucleotide sequence alignment of genes encoding polyphemusins. The genes encoding PM I and PM III were identified in the *Limulus polyphemus* whole genome shotgun sequence (GenBank: AZTN01102408.1 and AZTN01052275.1, respectively). Gaps in the sequences are shown as dashes. Putative exon and intron boundaries are indicated by black arrows. The deduced amino acid sequences of coding region are shown as a colored one-letter code: Signal peptide (orange), mature peptide (red), and anionic propiece (green). The yellow triangle points at the deletion cause a reading frame shift in the sequence of polyphemusin III. Putative prepropolyphemusin III amino acid sequence was translated from corresponding DNA considering that deletion of guanine nucleotide did not occur.

Nucleotide sequence alignment of genes encoding polyphemusins PM I and PM III in the genome of the horseshoe crab *Limulus polyphemus* showed that both peptides had the same length, but PM III involved four amino acids substitutions (W3G, Y14F, R15Q, K16R) compared with PM I (Figure 1). Noteworthy, a single nucleotide deletion was detected in the amidation signal GKR site located between the mature polyphemusin III and anionic propiece sequences. The deletion of the guanine nucleotide position appears to cause a shift of the open reading frame, however, the sequencing error by the *Limulus polyphemus* genome sequencing consortium also cannot be excluded. The aim of this study was to investigate the biological activities of a novel polyphemusin. 

### 2.2. Expression and Purification of the Recombinant Peptides

To improve the yield of the recombinant polyphemusins and tachyplesins after expression in *E. coli* and to facilitate their purification, the peptides were obtained as a part of the fusion proteins with the *N*-terminal octahistidine tag and the modified thioredoxin A (M37L). Following cell harvesting, sonication, preparative centrifugation of the cell lysate, affinity chromatography, and specific CNBr cleavage of the fusion proteins, the target peptides were purified by reversed-phase high-performance liquid chromatography (RP-HPLC) in a linear gradient of acetonitrile. MALDI mass spectrometry analysis of the main fractions (Figures S1 and S2) showed that the measured monoisotopic *m/z* matched well with the calculated molecular masses of protonated ions [M+H]^+^ of corresponding peptides (Table 1).

### 2.3. Antimicrobial Activity

The antimicrobial activity of PM III, its isoforms, and tachyplesins was determined by measuring their minimum inhibitory concentrations (MICs) against Gram-positive and Gram-negative bacteria using broth microdilution assay (Table 2). PM III displayed antimicrobial activity in a range of 0.25–2 μM against all strains except *S. aureus* ATCC 29213. However, compared to PM I, TP I, and TP III peptides, PM III demonstrated a 4-fold reduction in activity against the Gram-negative strains *E. coli* ML35p and *K. pneumoniae* (CI 287), and the Gram-positive strain *S. aureus* 209P. PM III had a similar potency against Gram-positive bacteria *B. subtilis* B-886 and *M. luteus* B-1314, and Gram-negative *P. aeruginosa* PAO1 compared to PM I and PM II. Moreover, identical activities were registered against *P. aeruginosa* PAO1 with MIC 0.5 μM among all tested peptides. All the peptides showed markedly reduced activities against *S. aureus* strain ATCC 29213 compared to other strains, with an 8-fold reduction in antimicrobial activity for polyphemusins and at least a 16-fold reduction for tachyplesins compared to *S. aureus* 209P. All tachyplesins demonstrated similar activities, with MICs ranging from 0.06 to 2 μM, except that measured against a low sensitive strain *S. aureus* ATCC 29213. MICs ranges determined for PM I (0.06–0.5 μM) and PM II (0.03–1 μM) were quite similar, with the above exception of that determined for *S. aureus* ATCC 29213. Compared to tachyplesins, PM I and PM II had, overall, higher or identical antibacterial effects.

### 2.4. Cytotoxic Effects on Human Cells

Cytotoxic activities of PM III, its isoforms, and tachyplesins against seven human cell lines were evaluated. Four cancer (HL-60 acute promyelocytic leukemia, HeLa cervix adenocarcinoma, SK-BR-3 breast adenocarcinoma, A549 lung carcinoma), one transformed (HEK 293T transformed human embryonic kidney), and two normal human cell lines (HEF human embryonic fibroblasts and NHA normal human astrocytes) were used for these experiments. Cytotoxicity was measured by incubating the cells with serial dilutions of PM I, PM II, PM III, TP I, TP II, or TP III samples followed by MTT assay after 48 h. The half-maximal inhibitory concentrations (IC_50_) were measured as the peptide concentration at which cell viability was reduced by 50% in comparison to untreated cells (Table 3).

Data analysis revealed that PM III had a potent cytotoxic activity against human cell lines. PM III was the most cytotoxically active among all tested peptides. IC_50_ values determined for PM III on cancer and transformed cell lines were in a range of 2.5–9.9 µM. Compared to PM III, the peptides PM I and PM II showed lower cytotoxic effects, with IC_50_ values ranging from 7.2 to 16.0 µM and from 7.2 to 17.3 µM, respectively. IC_50_ values for PM I or PM II tested against the same cancer, transformed, or normal cells were similar.

Tachyplesins turned out to be less toxic than polyphemusins. Among the investigated tachyplesins, TP III had the strongest cytotoxicity against cancer, transformed, and normal cell lines. The IC_50_ values determined for TP III on cancer and transformed cell lines were in a range of 5.0–27.5 µM, the IC_50_ values determined for the normal cells were of 14.1 µM for HEF and of 21.3 µM for NHA.

The leukemia cell line, HL-60, was the most sensitive to all investigated cytotoxic peptides. Moreover, HL-60 cells were two to three times more sensitive to PM III (IC_50_ = 2.5 µM) than to tachyplesins, PM I, and PM II. In contrast, the lowest sensitivity to cytotoxic peptides was observed for the SK-BR-3 cell line with IC_50_ values for all polyphemusins and tachyplesins ranging from 9.9 to 17.3 µM and from 27.5 to 34.0 µM, respectively.

The normal cell lines showed similar sensitivities to all polyphemusins: PM I (IC_50_ = 8.3 µM for HEF, IC_50_ = 14.5 µM for NHA), PM II (IC_50_ = 9.7 µM for HEF, IC_50_ = 18.3 µM for NHA), and PM III (IC_50_ = 7.0 µM for HEF, IC_50_ = 7.5 µM for NHA). The IC_50_ values determined for HEF and NHA cells were in the same range as for the cancer cell lines. Interestingly, the IC_50_ values of PM III determined for normal cells were several times higher than that for the most sensitive cell line, HL-60.

### 2.5. Hemolytic Activity

**Figure 2:**
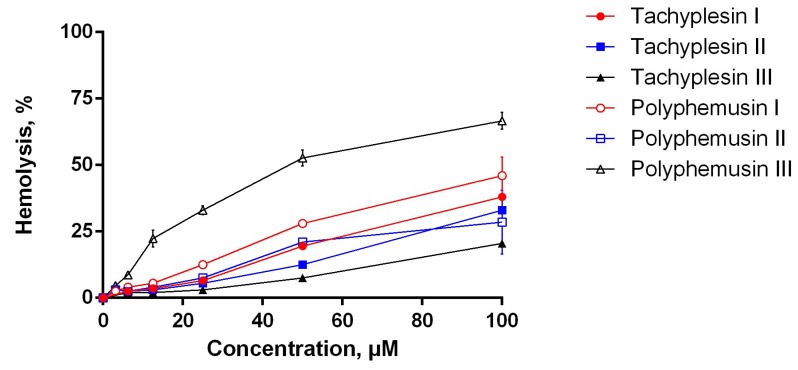
The hemolysis curve showing effects of polyphemusins and tachyplesins on human erythrocytes. The data represent the mean values ± SD for two independent series of triplicated experiments.

The assay for the hemolytic activity of PM III was performed to determine its effect on the integrity of red blood cells. At a concentration range of 3.125–100 µM, PM III had the highest hemolytic activity among all the peptides tested (Figure 2). The measured half-hemolysis concentration (HC_50_) value for PM III was of 46 µM. PM I and PM II were less toxic to erythrocytes and caused only ~45% and ~30% hemolysis, respectively, even at the maximal concentration of 100 µM. Tachyplesins also demonstrated low hemolytic effects: TP I, TP II, and TP III lysed 38%, 33%, and 20% of erythrocytes, respectively, at a maximal concentration of 100 µM.

### 2.6. Trypan Blue Assay for Dead Cells

According to the MTT-test, HL-60 was the most sensitive cell culture to PM III. Therefore, to investigate the mechanism of the PM III cytotoxicity, we first assessed a short-time membrane permeabilizing effect of PM III on HL-60 cells at different time points using the trypan blue exclusion assay. PM III was used at two final concentrations: Equal to IC_50_ (2.5 μM) and 2-fold exceeding IC_50_ (5 μM). The experiments were done at 15 min, 1 h, and 4 h following incubation with PM III.

**Figure 3:**
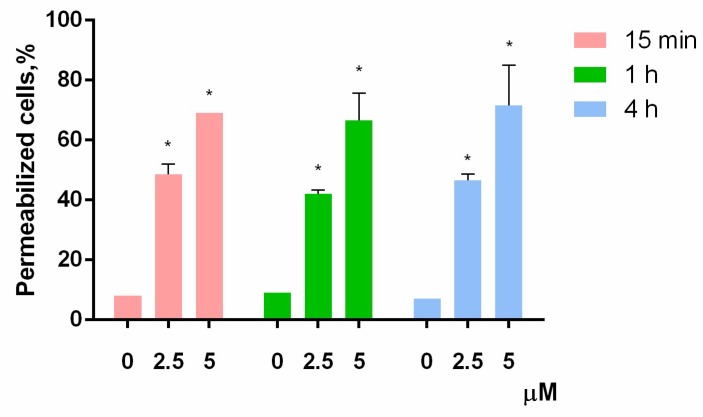
Trypan blue exclusion assay of HL-60 cell death after 15 min (red bars), 1 h (green bars), or 4 h (blue bars) of incubation with PM III (* *p* < 0.05 vs. the control sample of 0 µM for each time interval, respectively).

The proportion of dead HL-60 cells was virtually unchanged for all these time points at both concentrations used (Figure 3). In the negative control experiments, the dead cells ratio did not exceed 10%. In contrast, both concentrations of PM III were highly toxic to HL-60 cells, thus giving 42–48% of trypan-stained (dead) cells for 2.5 µM PM III for all incubation times, and 66–71% of dead cells for 5 µM PM III for all incubation times. Thus, the cytotoxic effect of PM III depended on its concentration, but not on the incubation times. We concluded, therefore, that PM III could induce cell death after 15 min of incubation, which is typical for necrotic cell death [22].

### 2.7. Mechanism of Cell Death

To further investigate the effect of PM III on human cells, we performed annexin V-FITC/propidium iodide double staining with subsequent flow cytometric analysis. Annexin V-FITC binds to phosphatidylserine, which is exposed on the outer leaflet of the cellular plasma membrane at the initial stages of apoptosis, whereas propidium iodide (PI) preferentially stains nuclei of dead cells. Therefore, a combination of annexin V-FITC and PI assays allows differentiation between early apoptotic and late apoptotic/necrotic cell populations. PM III was used at two final concentrations of 2.5 and 5 μM. In order to elucidate the predominant mechanism of the PM III action, we applied a widely used caspase inhibitor, Z-VAD-FMK [23].

**Figure 4:**
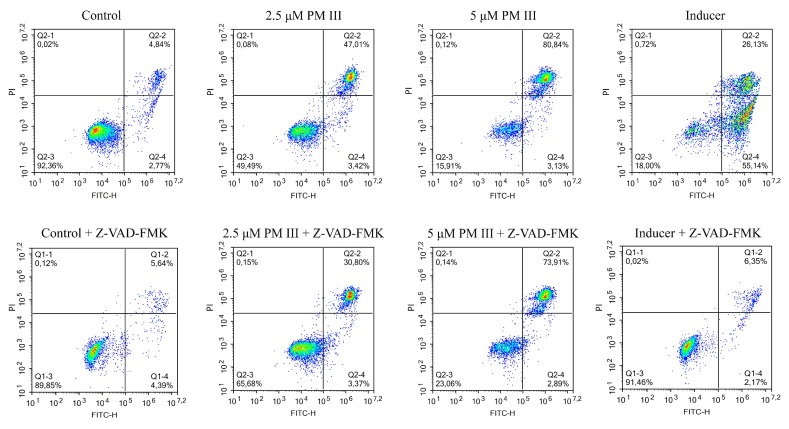
The flow cytometry results of annexin V-FITC/PI double staining of HL-60 cells after 4 h of incubation with PM III (at IC_50_ and 2xIC_50_) in the presence or absence of the caspase inhibitor, Z-VAD-FMK. Columns, from left to right: Control (untreated cells); 2.5 μM PM III; 5 μM PM III; 50 μM apoptotic inducer, campthotecin. Top row: No Z-VAD-FMK added, bottom row: 50 μM Z-VAD-FMK added. The results are presented as the percentage of viable (AV−PI−), apoptotic (AV+PI−), and late apoptotic/necrotic (AV+PI+) cells.

The results of HL-60 cells double staining, followed by the flow cytometry analysis are shown in Figure 4. The majority of the cells treated with 2.5 or 5 µM PM III were stained with both annexin V-FITC and PI and thus were either late apoptotic or necrotic. This was almost not affected by the presence of the apoptotic inhibitor, Z-VAD-FMK, following 4 h of incubation. In the absence of Z-VAD-FMK, > 40% of double-stained cells were detected for 2.5 µM PM III and >80% for 5 µM PM III. In the presence of Z-VAD-FMK, there were >30% of double-stained cells for 2.5 µM PM III, and > 70% for 5 µM PM III. In contrast, Z-VAD-FMK totally abrogated the effect of the 50 µM apoptotic inducer campthotecin that was used as a positive control.

### 2.8. Cell Membrane Integrity

**Figure 5:**
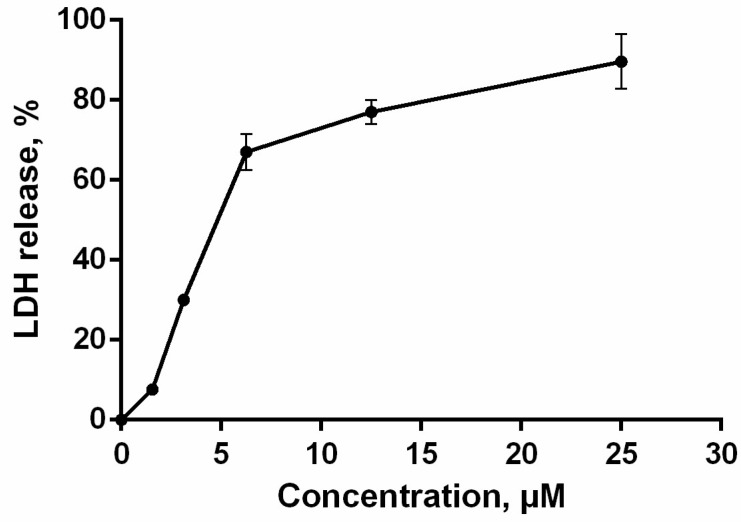
HL-60 cell membrane integrity after treatment with PM III. The data represent the mean values ± SD of two independent series of triplicated experiments.

The loss of membrane integrity, detected by trypan blue exclusion assay and annexin V-FITC/propidium iodide double staining, was also evaluated by lactate dehydrogenase (LDH)-release. The cytosolic enzyme LDH is released upon cell lysis and therefore can be a marker of cell integrity. The percentage of LDH-leakage from cells enables evaluation of direct cell lysis. The obtained results showed that PM III induced a concentration-dependent lysis in HL-60 cells within 1 h of incubation in a concentration range of 1.56–25 μM (Figure 5).

### 2.9. Gene Expression Profiling and Oncobox Pathway Analysis

**Figure 6:**
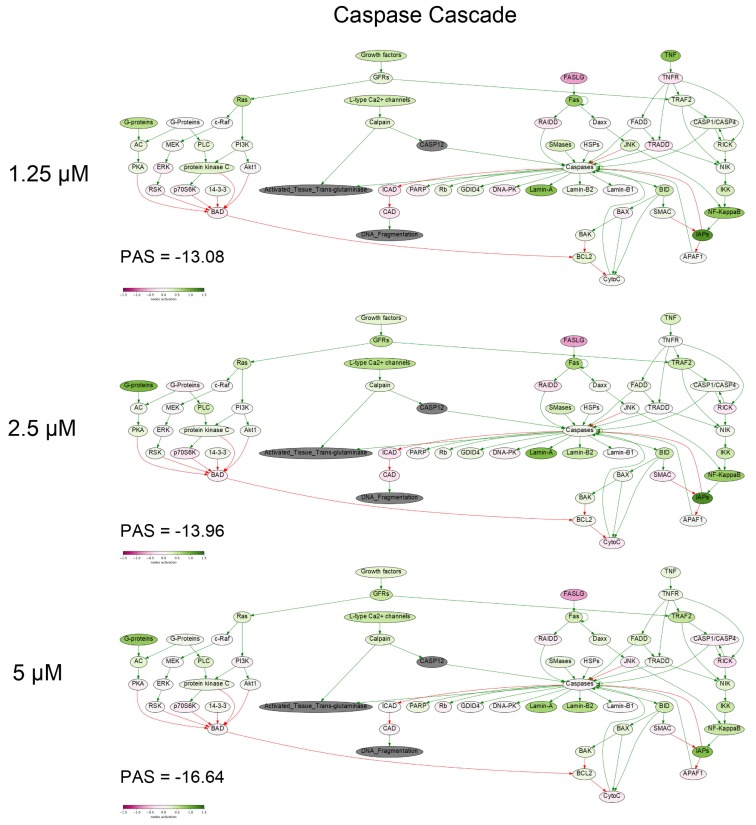
Caspase cascade pathway was inhibited in the PM III treated cells. The pathway was visualized using the Oncobox software (version 1.6.0-dev-1c6b124-modified). The pathway is shown as an interacting network, where green arrows indicate activation, red arrows—inhibition. The color depth of each node of the network corresponds to the logarithms of the case-to-normal (CNR) expression rate for each node, where “normal” is for intact cells, the scale represents an extent of up/downregulation.

**Figure 7:**
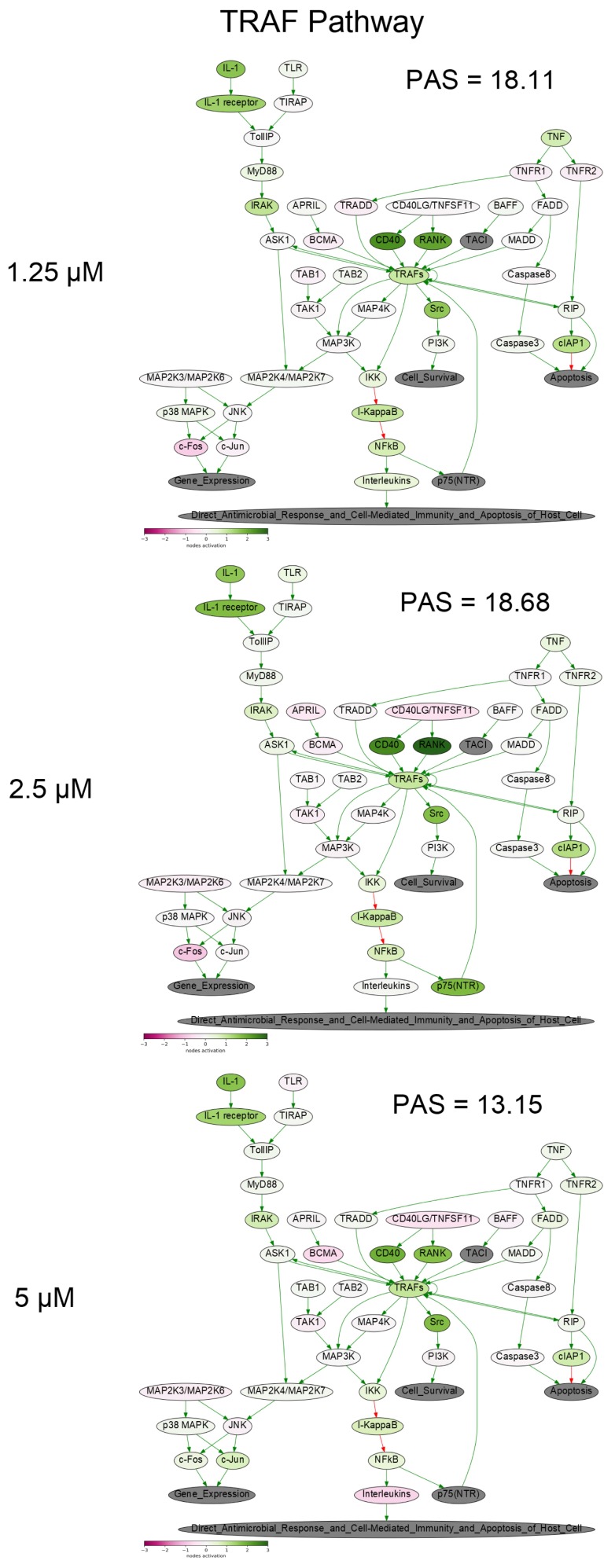
TRAF (tumor necrosis factor receptor (TNF-R)-associated factor) pathway was activated in the PM III treated cells. The pathway was visualized using the Oncobox software (version 1.6.0-dev-1c6b124-modified). The pathway is shown as an interacting network, where green arrows indicate activation, red arrows—inhibition. The color depth of each node of the network corresponds to the logarithms of the case-to-normal (CNR) expression rate for each node, where “normal” is for intact cells, the scale represents an extent of up/downregulation.

To get a deeper molecular insight into the mechanisms of cell death induced by PM III, we profiled gene expression in the PM III treated (1.25, 2.5, and 5 µM) and intact HL-60 cells. Gene expression profiles were further analyzed using the Oncobox bioinformatical platform to measure activation levels of 376 intracellular signaling pathways. We found a significant inhibition of the Caspase cascade (Figure 6), while the TRAF (tumor necrosis factor receptor (TNF-R)-associated factor) pathway was strongly activated (Figure 7) following the PM III treatment. An additional line of evidence for the non-apoptotic mechanism of the PM III mediated activity was provided by the PTEN pathway. It has been reported that PTEN most frequently had a positive connection to apoptosis [24]. The incubation with PM III strongly downregulated the PTEN signaling. The activation levels of all signaling pathways profiled in this study and case-to-normal ratios for the respective genes are shown in Supplementary Table S1. Taken together, these facts rather argue for a non-apoptotic mode of the PM III action.

We observed a number of molecular pathways promoting survival of the PM III treated cells. For example, the Akt and p38 signaling pathways were sequentially overactivated by increasing concentrations of PM III, thus probably representing the cellular pro-survival response on the PM III cytotoxic activity. Interestingly, we also found a number of immunity-linked pathways, namely Interferon response, Il-2, Il-6, and Il-10 pathways upregulated in most of the PM III-treated cell cultures (Table S1).

## 3. Discussion

In this study, for the first time to our knowledge, the antimicrobial peptide PM III was obtained in a bacterial expression system and studied. We examined its cytotoxic potential on bacteria and human cells. We studied PM III activities in comparison with its isoforms: PM I, PM II, and homologous horseshoe crab peptides, tachyplesins. Assessment of the antimicrobial activity of PM III evidenced that its MICs ranged from 0.25 to 2 μM (except that measured against the low sensitive strain, *S. aureus* ATCC 29213). Compared to the antimicrobial activities of PM I and PM II, the activity of PM III was either the same or lower against the bacterial strains tested.

Our experimental data for the obtained recombinant PM I are in line with those obtained earlier for synthetic PM I against Gram-negative and Gram-positive bacteria (with reported MICs not exceeding 1 μM) [14,16,25]. However, several reports on antimicrobial activities of natural and synthetic peptides TP I, TP II, PM I, and PM II demonstrated MICs higher than 1 μM [11,12]. In our study, the MICs of the recombinant tachyplesins did not exceed 2 μM.

PM III also caused half-maximal inhibition of human cell viability in the concentration range of ~2–10 μM. The PM III IC_50_ values were lower than those determined for PM I and PM II. We speculate that the higher cytotoxicity of PM III might be due to its higher hydrophobicity. In comparison to other tested peptides, the retention time of PM III measured during RP-HPLC indicated its higher hydrophobicity (Table 1). These data were congruent with the study results obtained with analogs of the synthetic amphipathic peptide V13K displaying antimicrobial and antitumor activities. Increased hydrophobicity of these analogs caused higher cytotoxic effects [26]. Previously published works showed a correlation between the hydrophobicity of peptides and their affinity to lipid bilayers in general [27,28,29,30]. Cell membranes contain different phospholipids, which sustain the amphipathic features due to the formation of hydrophilic and hydrophobic domains in the membrane structure. Well-known differences between the composition of bacterial and mammalian cell membranes provide the evidence for selectivity of the peptides’ action. Bacterial membranes predominantly consist of phosphatidylserine, phosphatidylglycerol, and cardiolipin having a net negative charge [31]. Tumor cell bilayers are also enriched in anionic molecules, such as phosphatidylserine, sialylated gangliosides, *O*-glycosylated mucins, and heparin sulfate. In contrast, zwitterionic phospholipids—phosphatidylcholine, phosphatidylethanolamine, and sphingomyelin—are abundant in the cytoplasmic membranes of non-cancer mammalian cells [32]. Electrically neutral normal mammalian membranes prevent their recognition by positively charged antimicrobial peptides, and binding to these membranes is enabled by hydrophobic interactions [33]. Thus, modulation of hydrophobicity of antimicrobial peptides, and also peptoid molecules, may be followed by non-specific interaction with erythrocytes as it was shown in previously published studies [30,34,35,36]. It was also revealed that antimicrobial peptides require optimal hydrophobicity levels to exhibit antimicrobial activity and retain selectivity [37]. Suboptimal high hydrophobic properties may not only result in increased toxicity to erythrocytes, but even in a reduction of antibacterial activity [37,38,39]. Several protegrin-1 synthetic analogs with different hydrophobicities were characterized earlier [40]. The analog with substitutions of valine by leucine that was more hydrophobic than naturally occurring protegrin-1 and the analog with substitutions of valine by fluorinated leucine had the most hydrophobic properties than other tested peptides. A highly hydrophobic protegrin-1 analog with fluorinated leucine was shown to be less potent against bacterial strains than naturally occurring protegrin-1. In contrast, the analog with leucine without fluorination exhibited the most pronounced antibacterial activity among all tested peptides. This demonstrates that the susceptibility of bacteria to peptides strongly depends on hydrophobicity levels [40]. These data are in line with the reduced antibacterial and increased hemolytic activities of the PM III peptide as compared to other tested peptides. The hemolytic activity of PM III determined in this study (HC_50_ = 46 µM) was rather high, but the HC_50_ value of PM III was 18-fold higher than its IC_50_ measured for the HL-60 cell line and ~5–7-fold higher than its IC_50_ measured for other cancer cell lines. Therefore, half-hemolysis concentration and IC_50_ effective concentration were in different ranges.

In this study, we obtained and studied the recombinant peptides. Despite the absence of naturally occurring post-translational *C*-terminal amidation, the recombinant polyphemusins demonstrated both antibacterial and cytotoxic activities. Our data obtained here for PM II matched well with the previously published results [9]. In this study, IC_50_ values for the recombinant PM II measured on human cancer, transformed, and normal cell lines were in a range of 7–18 μM. In the previous report, IC_50_ for synthetic PM II with the amidated *C*-terminal residue measured against leukemic and normal mononuclear cells ranged from 6 to 13 μM [9]. Furthermore, PM II caused a decrease of tumor cells’ viability with an IC_50_ of 6–13 μM, while the IC_50_ determined on mononuclear cells was 13 μM, indicating a general absence of selectivity towards tumor cells [9]. Similarly, we determined the IC_50_ for PM II on tumor cell lines, which ranged from 7 to 17 μM, while the IC_50_ determined for human embryonic fibroblasts and normal human astrocytes was 7 and 18 μM, respectively.

Previous studies revealed that TP I exhibited cytotoxic activity against tumor cells, with IC_50_ values ranging from 6 to 30 μM [9,41]. For example, in the case of glioma stem cells, IC_50_ values for synthetic TP I with the *N*-terminal acetylation and *C*-terminal amidation were ~10–20 μM, depending on incubation time [41]. Here, we showed that the recombinant non-amidated TP I had an IC_50_ ranging from 5 to 30 μM for different human tumor cells. The IC_50_ values for human embryonic fibroblasts and normal human astrocytes were 13 and 25 µM, respectively. Comparison with previously published studies indicated similar IC_50_ values for the normal cells. For the synthetic amidated TP I, previously measured IC_50_ for normal mononuclear cells was 15 μM [9]. The IC_50_ value for the naturally occurring TP I on monkey epithelial kidney cells (MA-104) was ~23 μM [42].

Suspension culture properties of HL-60 cells are supposed to be at the bottom of high sensitivity to polyphemusins and tachyplesins. In the previous report, the naturally occurring TP I halved the viability of HL-60 cells at ~10 μM [43]. For the synthetic amidated TP I and PM II peptides, the IC_50_ of 6–16 μM was previously observed on the suspension leukemia cell lines [9].

In this study, using trypan blue exclusion assay at different time points, we showed that the cytotoxic effect of PM III may develop in a relatively short time of 15 min of incubation. Furthermore, based on the data obtained by annexin V-FITC/PI double staining with subsequent flow cytometry, we found that PM III caused the cytotoxic effect most probably without involvement of apoptosis, and that the cytotoxic effect was not affected by the caspase inhibitor Z-VAD-FMK. The previous findings on PM II are in good agreement with our results, as the authors proposed Z-VAD-FMK-independent necrotic-like death of leukemia cells treated with PM II [8,9]. The loss of membrane cell integrity was also demonstrated by LDH-release assay. PM III induced lysis of cells followed by LDH leakage in a concentration-dependent manner within 1 h of incubation. Profiling of the gene expression and analysis of intracellular signaling pathways in human HL-60 cells incubated with PM III also supports the concept that peptide-mediated cell death is implemented likely without induction of apoptosis. In particular, there were no major pro-apoptotic signaling pathways activated by PM III (Table S1). Pathway analysis revealed inactivation of the entire caspase cascade pathway (Figure 6), which means that relative expression levels of most genes involved in this pathway are lower than in the control samples. We did not observe an increased expression for either p53 or its targets, such as TP53AIP1, PMAIP1, BAX, and BCL2. Expressions of these genes were, in most cases, below the normal levels (Table S1. Sheet “CNR”). Thus, we conclude that there were no detected signs of p53 activation linked with the PM III administration.

Summarizing all the above, in this study, PM III revealed the most significant cytotoxic effects against human cell cultures. It had a stronger cytotoxicity towards human leukemia HL-60 cells, as compared with the cytotoxic effect of other peptides tested against HL-60 in this study. Finally, we found that exposure of human leukemia cells to PM III not only caused cytotoxicity, but also mediated an intrinsic immune reaction via enhanced Interferon, Il-2, Il-6, and Il-10 signaling. This immunomodulatory feature of PM III suggests that it may be an attractive target for further biomedical screenings for future drug development.

## 4. Materials and Methods

### 4.1. Cell Lines and Culture Conditions

The following tumor and transformed cell lines were used in this study: HL-60 (acute promyelocytic leukemia), HeLa (human cervix adenocarcinoma cells), SK-BR-3 (human breast adenocarcinoma cells), A549 (human lung carcinoma cells), HEK 293T (transformed human embryonic kidney cells), HEF (human embryonic fibroblasts), and NHA (normal human astrocytes) were used as normal cell lines. Tumor and transformed cell lines were obtained from the American Type Culture Collection (ATCC; www.atcc.org). Primary cells HEF (normal human embryonic fibroblasts derived from human embryonic stem cells) and NHA (normal human astrocytes derived from human fetal brain tissue) were obtained and cultured according to the corresponding protocol [44,45]. Briefly, cells were cultured in DMEM/F12 (1:1) or RPMI-1640 medium containing 10% fetal bovine serum (Invitrogen, USA) at 37 °C in the atmosphere containing 5% CO_2_ and 95% air according to standard mammalian tissue culture protocols and using a sterile technique. All cell lines were tested by using the LookOut® *Mycoplasma* PCR Detection Kit (Sigma-Aldrich, St. Louis, MO, USA) according to the manufacturer’s protocol and found to be free of *Mycoplasma* infection.

### 4.2. Peptides

All the peptides were obtained by heterologous expression in *E.coli* with subsequent two-step purification as previously described for producing of β-hairpin peptides [46,47,48]. The recombinant plasmids for expression of the peptides were constructed with the use of pET-based vector as described previously [46]. The peptides were expressed in *E. coli* BL21 (DE3) cells as fusion proteins that included an octahistidine tag, the TrxL carrier protein (*E. coli* thioredoxin A with the Met37Leu mutation), a methionine residue and the mature polyphemusin I (GenBank: 14215.1), polyphemusin II (GenBank: 14216.1), polyphemusin III, tachyplesin I (GenBank: P14213.2), tachyplesin II (GenBank: P14214.2), or tachyplesin III (GenBank: P18252.1). The polyphemusin III primary structure was deduced from the whole genome shotgun sequence of *Limulus polyphemus* (GenBank: AZTN01052275.1). Oligonucleotides were designed based on *E. coli* codon usage bias. *E. coli* BL21 (DE3) cells transformed with the constructed plasmids were grown up to OD_600_ 1.0 and then were induced with 0.2 mM IPTG. The induction was performed at 30 °C for 5 h under stirring with a shaking speed of 220 rpm. The peptides’ purification included immobilized metal affinity chromatography (IMAC) of cell lysate, CNBr cleavage of the fusion proteins, and reversed-phase high-performance liquid chromatography (RP-HPLC) as described previously; the peptides had a purity of at least 98% [46]. The RP-HPLC fractions were dried in vacuo, dissolved in water, and analyzed by MALDI-MS (Bruker Daltonics, Bremen, Germany). The obtained non-amidated recombinant analogs of natural polyphemusin I, polyphemusin II, polyphemusin III, tachyplesin I, tachyplesin II, and tachyplesin III were analyzed by automated microsequencing with the use of the Procise cLC 491 Protein Sequencing System (PE Applied Biosystems, Foster City, CA, USA). The peptides’ concentrations were estimated based on near-UV absorbance measurement and calculated with the use of extinction coefficients.

### 4.3. Antimicrobial Assay

Bacterial test cultures were grown in the Mueller-Hinton (MH) medium at 37 °C to mid-log phase and then diluted with the 2 x MH medium supplemented with 1.8% NaCl. 50 µL of the bacterial suspension with a final cell concentration of 10^6^ CFU/mL were added to aliquots of 50 µL of the peptide solutions and serially diluted with sterilized 0.1% bovine serum albumin in 96-well flat-bottom polystyrene microplates (Eppendorf, Hamburg, Germany). Bacterial cells were incubated for 24 h at 37 °C and 900 rpm on the plate thermoshaker (Biosan, Riga, Latvia). The minimum inhibitory concentrations (MIC) were determined as the lowest peptide concentration that prevented growth of a test microorganism observed as visible turbidity. The results were expressed as median values of three independent triplicated experiments. Assessment of antimicrobial activities of the recombinant PM III, other polyphemusins, and tachyplesins was conducted against Gram-negative (*Escherichia coli* ML-35p, *Klebsiella pneumoniae* (clinical isolate, CI 287), *Pseudomonas aeruginosa* PAO1) and Gram-positive (*Staphylococcus aureus* ATCC 29213, *Staphylococcus aureus* 209P, *Bacillus subtilis* B-886, *Micrococcus luteus* B-1314) bacteria.

### 4.4. Cytotoxic Activity Assay

The colorimetric 3-(4,5-dimethylthiazol-2-yl)-2,5-diphenyltetrazolium bromide (MTT) dye reduction assay was used to determine the cytotoxicity of the polyphemusins and tachyplesins. Cells (3 × 10^3^–6 × 10^3^ per well) were placed into 96-well plates in Dulbecco’s modified Eagle’s medium (DMEM/F12) supplemented with 10% fetal bovine serum (FBS). After incubation in the atmosphere containing 5% CO_2_ and 95% air at 37 °C overnight, the media were discarded, and polyphemusins or tachyplesins solutions were added to the cell cultures up to final concentrations of 2.5, 5, 10, 25, and 50 μM in a final volume of 0.1 mL DMEM/F12 with 10% FBS. HL-60 cells (4 × 10^4^ in 50 μL) were added to two-time serial dilutions of polyphemusins solutions at a final concentration of 1.25, 2.5, 5, 10, 25, and 50 μM in 50 μL RPMI-1640 with 10% FBS. 48 hours later, 20 μL of MTT (5 mg/mL) was added into each well and the plates were incubated for 3 h at 37 °C. The plates with HL-60 cells were centrifuged for 10 min at 300 g. The media were discarded and 0.1 mL of dimethyl sulfoxide and isopropanol mixture at a ratio of 1:1 (v/v) was added to each well to dissolve the crystallized formazan. The absorbance at 570 nm was measured by a microplate reader (Eppendorf, Hamburg, Germany).

### 4.5. Hemolytic Activity Assay

Testing of the hemolytic activity of polyphemusins and tachyplesins was performed using fresh human red blood cells (hBRC). Permeabilization of erythrocyte cytoplasmic membrane results in lysis followed by the release of hemoglobin. hBRC were washed three times with phosphate buffered saline (PBS: 10 mM Na_2_HPO_4_, 1.76 mM K_2_HPO_4_, pH 7.4, containing 173 mM NaCl, and 2.7 mM KCl). Two-fold serial dilutions of the peptide solutions were added to 50 μL aliquots of hRBC in the 96-well microplate. The final hBRC concentration was of 4% (v/v) in each well, and the final volume of suspension was 100 μL. After incubation of suspensions for 1.5 h at 37 °C under stirring at 1000 rpm, centrifugation of plates was done at 700 *g* for 5 min. The supernatant 50 μL aliquots were then transferred into flat-bottomed 96-well microplates. The absorbance at 405 nm was measured in a microplate reader (Eppendorf, Hamburg, Germany), allowing the detection of hemoglobin release. 0.1% Triton X-100 caused a complete lysis of erythrocytes, thus hBRC treated with Triton X-100 were used as a positive control. As PBS did not cause lysis, hBRC in PBS served as a negative control. The percentage of hemolysis was calculated using the following Equation (1):

Hemolysis (%) = (OD_405_ sample − OD_405_ 0% lysis control)/(OD_405_ 100% lysis control − OD_405_ 0% lysis control) × 100% (1)

The quantitative data resulted from two series of experiments were represented as average means with standard deviations. The experiments were carried out using hBRC obtained from human blood samples of independent donors.

### 4.6. Trypan Blue Exclusion Assay

Polyphemusin III was diluted in non-supplemented RPMI medium and incubated with 2.5 × 10^5^ HL-60 cells in a volume of 100 μL. After 15 min, 1 h, or 4 h of incubation with 2.5 or 5 μM polyphemusin III, proportions of viable cells were counted in an automated cell counter (Thermo Fisher Scientific, Waltham, MA, USA) using sterile-filtered 0.4% trypan blue solution. The quantitative data were represented as average means with standard deviations (±SD) obtained from three independent experiments.

### 4.7. Annexin V-FITC/Propidium Iodide Double Staining and Flow Cytometry

Cell death analysis with fluorescein isothiocyanate conjugated annexin V (annexin V-FITC)/propidium iodide double staining and dead cells counting with flow cytometry were performed 4 h after addition of polyphemusin III up to a final concentration of 2.5 or 5 μM. The Annexin V-FITC Apoptosis Detection Kit (BD Biosciences, Franklin Lakes, NJ, USA) was used according to the manufacturer’s protocol on a NovoCyte flow cytometer (ACEA Biosciences, San Diego, CA, USA). Each experiment was performed in triplicate. The apoptosis inducer camptothecin (Sigma-Aldrich, St. Louis, MO, USA) and the general caspase inhibitor, Z-VAD-FMK (BD Biosciences, Franklin Lakes, NJ, USA), were used. All flow cytometry experiments were performed twice.

### 4.8. Lactate Dehydrogenase (LDH)-Release Assay

The LDH-release assay (CytoTox 96 Non-Radioactive Cytotoxicity Assay; Promega Corporation, Madison, WI, USA) was performed according to the manufacturer’s protocol. Suspension HL-60 cells (4 × 10^4^) were seeded into a 96-well plate in triplicate and incubated for 1 h with various concentrations of polyphemusin III at 100 μL per well in RPMI serum-free medium. Untreated cells served as a control for spontaneous LDH-release and cells treated with 1% Triton X-100 were used as a control for maximal LDH-release. Absorbance was measured at 490 nm using a microplate reader (Eppendorf, Hamburg, Germany). Percent LDH-release was calculated using the following Equation (2):

[(experimental LDH-release − spontaneous LDH-release)/(maximal LDH-release − spontaneous LDH-release)] × 100(2)

The dose response curve shown is an average of two independent experiments.

### 4.9. Statistical Analysis

The GraphPad PRISM 6.0 software (GraphPad Software Inc., San Diego, CA, USA) was used for statistical analysis; the values of *p* < 0.05 were considered statistically significant.

### 4.10. Gene Expression Profiling and Functional Annotation

RNA extraction was performed immediately before preparation of sequencing libraries using a QIAGEN RNeasy Kit (Qiagen, Venlo, Netherlands) following the manufacturer’s protocol. Then, the RNA Integrity Number (RIN) was measured using an Agilent 2100 bio-Analyzer. Agilent RNA 6000 Nano or Qubit RNA Assay Kits were used to measure RNA concentration. A KAPA RNA Hyper with RiboErase (KAPA Biosystems, Wilmington, MA, USA) kit was used for further depletion of ribosomal RNA. For library preparations, we used the Ovation® Universal RNA-Seq System and 1-96 KAPA HyperPrep Kit according to the manufacturer’s recommendations. Different adaptors were used for multiplexing samples in one sequencing run. Library concentrations and quality were measured using a Qubit ds DNA HS Assay kit (Life Technologies, Carlsbad, CA, USA) and Agilent Tapestation (Agilent, Santa Clara, CA, USA). RNA sequencing was performed using Illumina HiSeq 3000 equipment for single-end sequencing, 50 bp read length, for approximately 30 million raw reads per each sample. The data quality check was done on an Illumina SAV. De-multiplexing was performed with an Illumina Bcl2fastq2 v 2.17 program.

RNA sequencing FASTQ files were processed with a STAR aligner [49] in the “GeneCounts” mode with the Ensembl human transcriptome annotation (Build version GRCh38 and transcript annotation GRCh38.89). Ensembl gene IDs were converted to HGNC gene symbols using the Complete HGNC dataset (https://www.genenames.org/, database version from 2017 July 13). In total, expression levels were established for 36596 annotated genes with corresponding HGNC identifiers.

The SABiosciences knowledge base was used to determine structures of intracellular molecular pathways as described previously [50,51]. We applied the original Oncobox algorithm [50] for functional annotation and visualization of the primary expression data and for calculating pathway activation strength (PAS) scores and case-to-normal ratios (CNRs). CNRs were calculated as the ratios of expression levels of a gene in the experimental samples to average expression levels in the control samples. PAS values can be both positive and negative, being indicative of up- or downregulation compared to controls.

## 5. Conclusions

We investigated the properties and mechanism of cytotoxicity of the novel peptide PM III from the horseshoe crab *Limulus polyphemus*. It had the strongest cytotoxicity against human cells among all the peptides interrogated in this study and, despite its high hemolytic activity, may be regarded as a potentially useful object for further studies on cytotoxicity towards tumor cells. We showed here that the cytotoxic activity of PM III is based on fast disruption of the cell membrane.

## Figures and Tables

**Table 1:** Primary structure and physicochemical characteristics of polyphemusins and tachyplesins.

Peptide	Amino Acid Sequence ^1^	Calculated [M+H]^+^ Monoisotopic Mass, Da ^2^	Measured Monoisotopic *m/z* Value ^3^	Net Charge	RP-HPLC Retention Time ^4^, min	Recombinant Peptide Final Yield, mg/L
PM I	RRWCFRVCYRGFCYRKCR	2454.18	2453.97	+7	35.5	2.4
PM II	RRWCFRVCY**K**GFCYRKCR	2426.18	2426.19	+7	35.4	1.5
PM III	RR**G**CFRVCYRGFC**FQR**CR	2309.09	2309.01	+6	36.2	5.9
TP I	KWCFRVCYRGICYRRCR	2264.10	2264.25	+6	35.5	3.4
TP II	**R**WCFRVCYRGICYR**K**CR	2264.10	2264.14	+6	35.9	3.0
TP III	KWCFRVCYRGICYR**K**CR	2236.09	2236.07	+6	35.5	3.0

^1^ Amino acid substitutions in PM II and PM III (as compared to PM I) and in TP II and TP III (as compared to TP I) are shown in bold. ^2^ Molecular masses were calculated by considering the presence of four Cys residues forming two disulfide bonds and ^3^ were determined experimentally using MALDI mass spectrometry. ^4^ Retention times were measured using semi-preparative reversed-phase high-performance liquid chromatography (RP-HPLC) on a C18 column with a linear gradient from 5 to 80% (v/v) of acetonitrile in water containing 0.1% trifluoroacetic acid (TFA) within 1 h.

**Table 2:** Antimicrobial activities of polyphemusins and tachyplesins.

Strain	Gram	MICs, μM					
		PM I	PM II	PM III	TP I	TP II	TP III
*E. coli* ML-35p	-	0.062	0.031	0.25	0.062	0.062	0.062
*K. pneumoniae* (CI 287)	-	0.5	0.5	2	0.5	1	0.5
*P. aeruginosa* PAO1	-	0.5	0.5	0.5	0.5	0.5	0.5
*S. aureus* ATCC 29213	+	4	4	16	8	8	16
*S. aureus* 209P	+	0.5	0.5	2	0.5	0.5	0.5
*B. subtilis* B-886	+	0.25	0.5	0.5	0.5	0.5	1
*M. luteus* B-1314	+	0.5	1	0.5	1	1	2

**Table 3:** The IC_50_ values measured for polyphemusins and tachyplesins on human cell lines.

Cell Line	IC_50_, μM					
	PM I	PM II	PM III	TP I	TP II	TP III
HL-60	7.2 ± 0.5	7.2 ± 0.5	2.5 ± 0.1	4.8 ± 0.3	5.6 ± 0.5	5.0 ± 0.4
HeLa	12.5 ± 0.9	16.4 ± 1.3	6.0 ± 0.2	24.2 ± 1.7	24.4 ± 3.0	13.7 ± 1.3
SK-BR-3	16.0 ± 0.6	17.3 ± 0.6	9.9 ± 0.1	30.1 ± 1.9	34.0 ± 1.3	27.5 ± 0.8
A549	8.8 ± 1.3	10.8 ± 1.9	7.3 ± 0.5	26.5 ± 1.2	28.1 ± 1.7	15.3 ± 1.0
HEK 293T	9.4 ± 1.5	11.3 ± 1.6	7.3 ± 0.4	23.5 ± 1.2	24.6 ± 1.7	19.7 ± 1.9
HEF	8.3 ± 0.7	9.7 ± 0.2	7.0 ± 0.4	13.0 ± 1.5	17.7 ± 1.2	14.1 ± 1.2
NHA	14.5 ± 1.6	18.3 ± 1.4	7.5 ± 0.5	24.7 ± 1.8	29.4 ± 1.7	21.3 ± 1.2

IC_50_ values are represented as the means ± standard deviations (SD) of at least three independent experiments.

## Supplementary Materials

The following are available online at http://www.mdpi.com/1660-3397/16/12/466/s1. Figure S1: MALDI-MS analysis of the recombinant (**A**) polyphemusin I, (**B**) polyphemusin II and (**C**) polyphemusin III. The experimental [M+H]^+^ monoisotopic *m/z* is presented in the picture. Figure S2: MALDI-MS analysis of the recombinant (**A**) tachyplesin I, (**B**) tachyplesin II and (**C**) tachyplesin III. The experimental [M+H]^+^ monoisotopic *m/z* is presented in the picture. Figure S3: Reversed-phase high-performance liquid chromatography (RP-HPLC) of the recombinant (**A**) polyphemusin I, (**B**) polyphemusin II and (**C**) polyphemusin III. RP-HPLC was performed with a linear gradient from 5 to 80% (v/v) of acetonitrile in water containing 0.1% TFA within 1 h. The fractions of the mature recombinant peptides are marked with arrows. Figure S4: Reversed-phase high-performance liquid chromatography (RP-HPLC) of the recombinant (**A**) tachyplesin I, (**B**) tachyplesin II and (**C**) tachyplesin III. RP-HPLC was performed with a linear gradient from 5 to 80% (v/v) of acetonitrile in water containing 0.1% TFA within 1 h. The fractions of the mature recombinant peptides are marked with arrows. Table S1: Pathway activation strength (PAS) and case-to-normal (CNR) values for unaffected and polyphemusin III-treated cells (at the peptide concentration of 1.25, 2.5, and 5 µM).
