# Supplementary material for: Cytotoxic Potential of the Novel Horseshoe Crab Peptide Polyphemusin III
Source: Mar Drugs. 2018 Nov 26;16(12):466. doi: 10.3390/md16120466 (PMC6315362; doi:10.3390/md16120466)
Supplement: Supplementary file 1 [file marinedrugs-16-00466-s001.zip › marinedrugs-392383-supplementary/Track REVISED marinedrugs-392383-Supplementary-File 1.docx]

**Supplementary Materials**

**Cytotoxic Potential of the Novel Horseshoe Crab Peptide Polyphemusin III**

Mariana B. Marggraf ^1^, Pavel V. Panteleev ^1^, Anna A. Emelianova ^1^, Maxim I. Sorokin ^2,3^,
Ilia A. Bolosov ^1^, Anton A. Buzdin ^1,2,3^, Denis V. Kuzmin ^1^ and Tatiana V. Ovchinnikova ^1,3,^*

^1^ M.M.Shemyakin & Yu.A.Ovchinnikov Institute of Bioorganic Chemistry, The Russian Academy of Sciences, Mikhluho-Maklaya str., 16/10, Moscow 117997, Russia; thpcb92@mail.ru (M.B.M.);
alarm14@gmail.com (P.V.P.); annaemelyan@gmail.com (A.A.E.); b_off2@mail.ru (I.A.B.);
buzdin@oncobox.com (A.A.B.); denisk@list.ru (D.V.K.); ovch@ibch.ru (T.V.O.);

^2^ Department of Bioinformatics and Molecular Networks, Omicsway Corp., Walnut, CA 91789, USA; sorokin@oncobox.com (M.I.S.);

^3^ I.M. Sechenov First Moscow State Medical University (Sechenov University), Moscow 119991, Russia;

***** Correspondence: ovch@ibch.ru; Tel.: +7-495-336-44-44


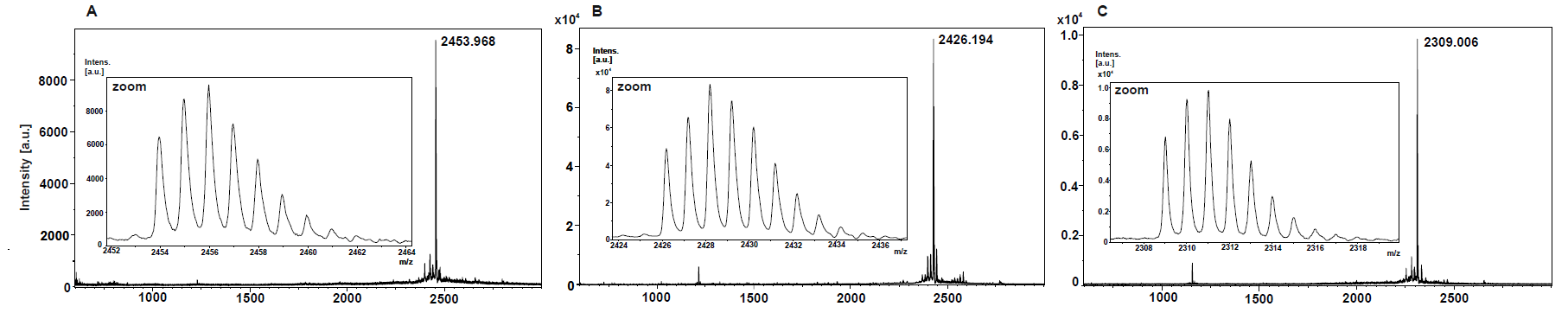


**Figure S1.** MALDI-MS analysis of the recombinant (**A**) polyphemusin I, (**B**) polyphemusin II and (**C**) polyphemusin III. The experimental [M+H]^+^ monoisotopic *m*/*z* is presented in the picture.


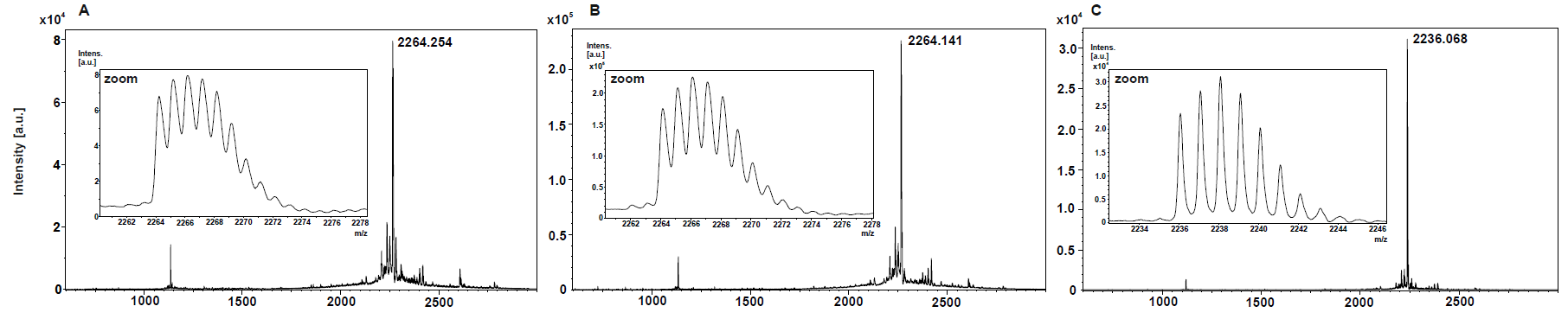


**Figure S2.** MALDI-MS analysis of the recombinant (**A**) tachyplesin I, (**B**) tachyplesin II and (**C**) tachyplesin III. The experimental [M+H]^+^ monoisotopic *m*/*z* is presented in the picture.


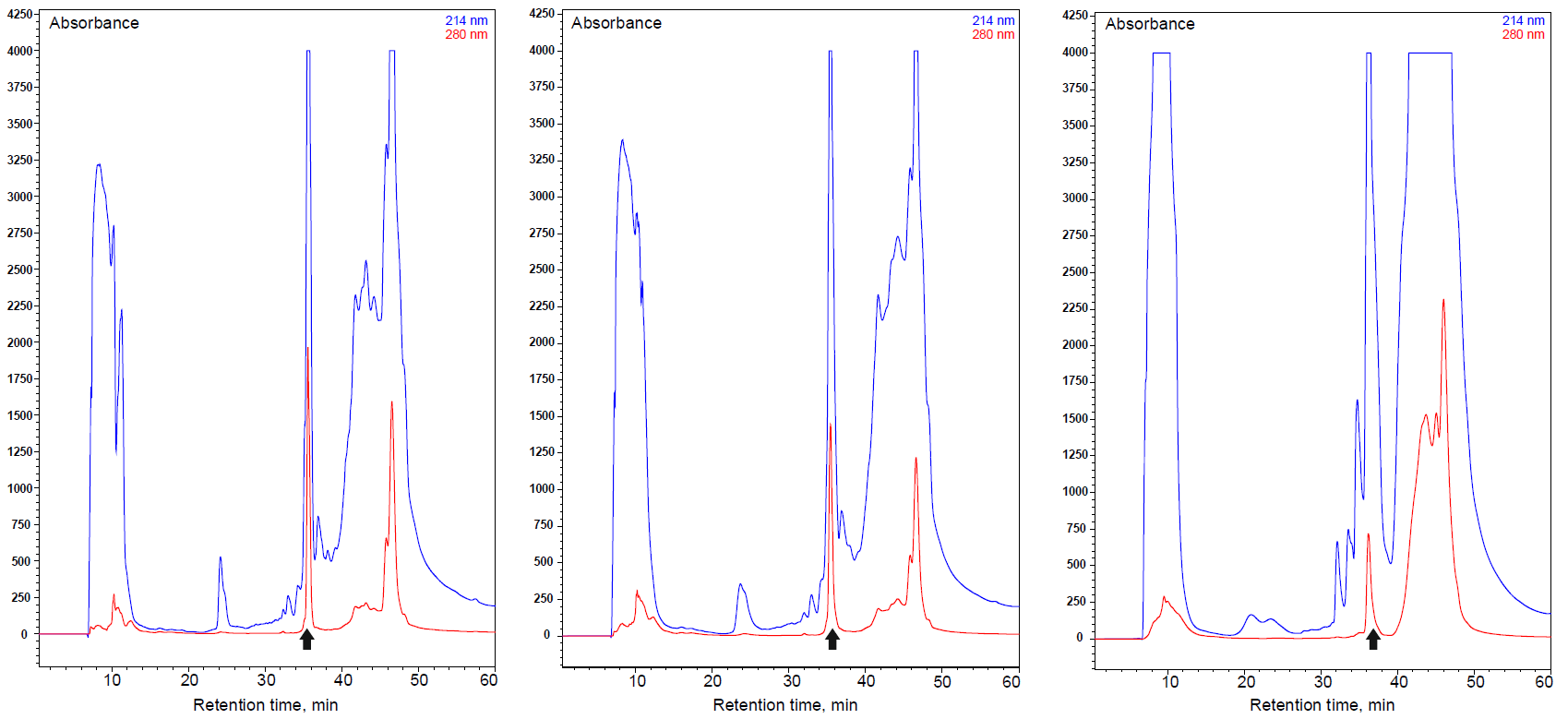


**Figure S3.** Reversed-phase high-performance liquid chromatography (RP-HPLC) of the recombinant (**A**) polyphemusin I, (**B**) polyphemusin II and (**C**) polyphemusin III. RP-HPLC was performed with a linear gradient from 5 to 80% (v/v) of acetonitrile in water containing 0.1% TFA within 1 h. The fraction of the mature recombinant peptides are marked with arrowes.


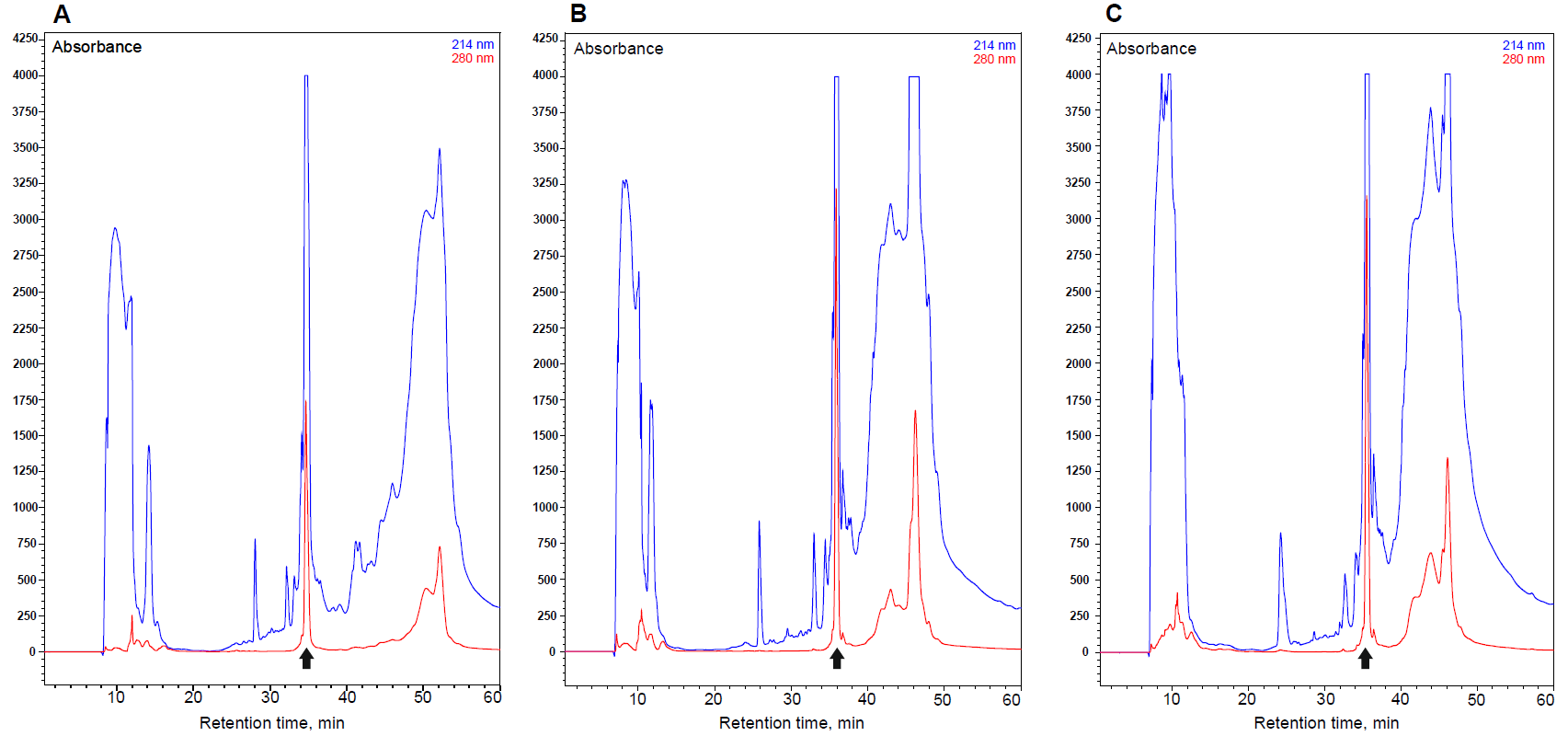


**Figure S4.** Reversed-phase high-performance liquid chromatography (RP-HPLC) of the recombinant (**A**) tachyplesin I, (**B**) tachyplesin II and (**C**) tachyplesin III. RP-HPLC was performed with a linear gradient from 5 to 80% (v/v) of acetonitrile in water containing 0.1% TFA within 1 h. Black arrows mark the fraction mature recombinant peptides.
